# Immunomodulatory biomaterials as a novel therapeutic platform for inflammatory bowel disease

**DOI:** 10.1177/17562848261463601

**Published:** 2026-07-14

**Authors:** Lydia Blacklock, Gordon Moran, Alvaro Mata, Amir Ghaemmaghami

**Affiliations:** NIHR Nottingham Biomedical Research Centre, Nottingham University Hospitals NHS Trust and The University of Nottingham, Nottingham, UK; NIHR Nottingham Biomedical Research Centre, Nottingham University Hospitals NHS Trust and The University of Nottingham, Nottingham, UK; School of Pharmacy, University of Nottingham, Nottingham, UK; Biodiscovery Institute, University of Nottingham, Nottingham, UK; Department of Chemical and Environmental Engineering, University of Nottingham, Nottingham, UK; School of Life Sciences, University of Nottingham, NG7 2RD, UK

**Keywords:** biomaterials, immunomodulation, inflammatory bowel disease, supramolecular assembly

## Abstract

Inflammatory bowel disease (IBD) is a chronic autoimmune condition of the gut caused by an inappropriate reaction towards commensal bacteria by immune cells that reside within the gut-associated lymphoid tissue. IBD is of rising concern due to increased global incidence with no outright cure. Patients have changeable response to standard therapeutic pathways that result in lifelong contact with healthcare systems. This narrative review aims to evaluate the therapeutic potential of immunomodulatory biomaterials for IBD as an alternative approach to current treatment options. A literature search was conducted using PubMed, Google Scholar and Web of Science databases. The primary aim was to identify pre-clinical, laboratory studies that investigated biomaterials in the presence of IBD models. Herein, we review the advent of biomaterials that demonstrate immunomodulatory capacity to directly alleviate chronic IBD pathology. We demonstrate the difference between natural and synthetic polymers as building blocks for biomaterials, such as hydrogels, microspheres and nanospheres, which can be functionalised based upon specific IBD inflammatory markers. Here, we assess examples of immunomodulatory biomaterials tested in IBD cellular, tissue and animal models as inflammation-targeted alternatives to current therapeutics. We also discuss the gaps for further research, from administration to scalability considerations to demonstrate the realistic use of immunomodulatory biomaterials for IBD in the clinic.

## Plain language summary

Immunomodulatory biomaterials for inflammatory bowel disease: using unique materials that are body-friendly to directly calm lifelong immune inflammation within the digestive tract

Inflammatory Bowel Disease (IBD) is a chronic condition that results in stomach cramps, diarrhoea, bloody stools and loss of appetite. IBD restricts patients’ day-to-day due to the unpredictability of symptoms. Usually, a mucus layer separates the good commensal gut bacteria from cells lining the intestines. In IBD the mucus layer is lost, and the cell layer becomes leaky which leads to the bacteria continually activating the immune system that resides underneath the cell layer to cause chronic inflammation and wounding of the gut lining. The number of people affected by the disease is increasing with major concern for the rate in young people which creates a lifelong interaction with the healthcare system. There is no drug that can cure IBD only alleviate the symptoms to a level that is manageable for daily activities. Furthermore, patients can often become unresponsive to their treatment and must embark on new therapeutic pathways or undergo major surgery to remove the affected tissue. The lack of treatments that address the inflammation at the site of action prevents patients being cured from the disease. This review paper analyses research into new treatment options in the form of biomaterials. Biomaterials are materials that can be in contact with the body and are often altered to prevent rejection. The use of biomaterials for IBD is of interest due to the ability to easily engineer features to specific markers of inflammation. There are still gaps in the research that must be addressed for biomaterials to be considered a credible alternative to conventional IBD therapeutics.

## Introduction

Inflammatory bowel disease (IBD) is a chronic autoimmune condition that affects approximately 7 million people globally. IBD takes two principal forms, ulcerative colitis (UC) and Crohn’s disease (CD).^1^ Intestinal inflammation manifests as a range of symptoms like chronic diarrhoea, abdominal pain and weight loss.^2^ This is due to environmental and genetic predisposition, which causes intestinal commensal bacteria to be detected as pathogens by the gut-associated lymphoid tissue (GALT), which leads to dysbiosis, sustained inflammation and remodelling of the intestines due to an overactive immune system.^3^

The intestines are the largest immunological organ with 10^12^ lymphoid cells per metre of human intestine. Ingestion of 130–190 g of dietary proteins every day, as well as the colonisation of the intestines with commensal bacteria (10^12^ microorganisms per gram of stool), would usually create a highly reactive immune environment towards countless foreign materials. However, the lymphoid cells in the intestines are specialised at being inflammation anergic and dampen reactions towards these food antigens, known as oral tolerance.^4^ Oral tolerance is a facet of peripheral tolerance and is hypothesised to have evolved as a self-tolerance mechanism to prevent hypersensitivity reactions to food and bacterial antigens.^4^ Oral tolerance is the mechanism to suppress immune responses against antigens that have already entered the body via oral administration and is maintained by the lymphoid cells present in the GALT.^4^ Oral tolerance induction is attributed to CD103+ dendritic cells detecting orally derived antigens and homing to mesenteric lymph nodes. The dendritic cells imprint antigenic detection to CD25+Foxp3+ induced suppressor T regulatory cells (Tregs), which return to the lamina propria to execute tolerogenic effects by inducing production of anti-inflammatory cytokines like Interleukin (IL)-10 and IL-4 from T cells. This process requires the presence of transforming growth factor-β (TGF-β) and retinoic acid. Furthermore, oral tolerance has a dose-dependent effect, with immune cell anergy or clonal deletion occurring in response to a high dose of antigen, whilst induction of suppressor Tregs occurs in response to low, repeated dose of antigen.^5^ Oral tolerance is integral for maintaining gut homeostasis and is continuously monitored by lymphoid cells located in the GALT.

**Figure 1.**
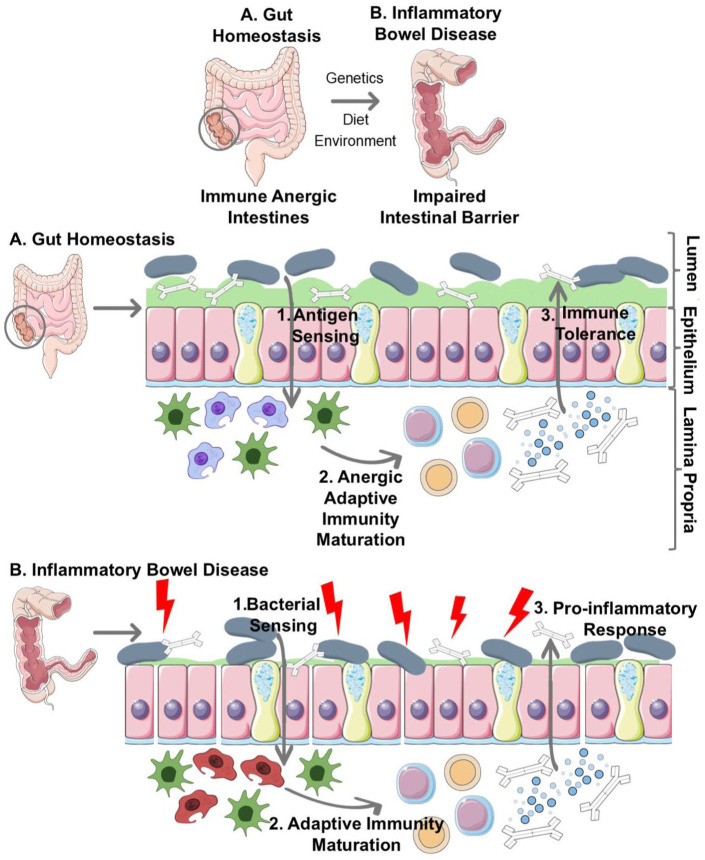
Schematic demonstrating the mechanism of IBD. The intestines experience a shift from an anergic (a) to a pro-inflammatory environment (b), due to antigen sensing of commensal bacteria. This results in an impaired intestinal barrier in the form of a leaky epithelium and reduced mucus layer, leading to the detection of commensal bacteria by innate immune cells. Source: Graphics adapted from Servier Medical Art (https://smart.servier.com), licensed under CC BY 4.0 (https://creativecommons.org/licenses/by/4.0/).

The GALT extends into the lamina propria as specialised tissue in the form of Peyer’s patches in the small intestine and isolated lymphoid follicles in both the small and large intestines. These connect to lymphoid organs via the underlying draining lymphatic vessels to facilitate adaptive immune cell maturation. The lamina propria is located beneath a monolayer of various intestinal epithelial cells (IECs) and is held together by strong tight junctions between cell types.^6^ Importantly, the goblet cells are specialised at secreting mucin proteins to form a mucus layer on top of the IECs to separate the commensal bacteria from the epithelium and underlying lamina propria (Figure 1). Due to multifactorial aetiology, IBD results in the breakdown of GALT homeostasis, allowing commensal bacteria to make direct contact with IECs, leading to a leaky epithelium and depletion of the mucus layer. This results in inappropriate detection of the commensal bacteria by the GALT, which causes a pro-inflammatory immune response (Figure 1). The millions of bacteria are continuously processed by the GALT, and a positive feedback loop of detection and inflammation is created that results in gut wounding.^7,8^

Clinicians prescribe IBD patients various treatment strategies to re-establish gut homeostasis and enter clinical remission. Although current therapies can alleviate symptoms, they may exert systemic side effects and fail to address structural changes in the gut lining. Moreover, most therapies reach a therapeutic ceiling of clinical response in the region of ~50%, leaving a considerable number of patients with refractory symptoms and poor quality of life.^9^ Alongside other autoimmune diseases, IBD poses a substantial burden on healthcare systems due to rising incidence.^10^ A particular concern is the increasing diagnosis of paediatric cases, which leads to prolonged therapeutic interventions and extended interactions with the healthcare system.^11^ This often means that patients face a lifetime of clinical monitoring to reduce debilitating symptoms. Thus, more research is necessary to identify novel drug candidates or modalities for therapeutic interventions to address the refractory nature of IBD. There are multiple strands of pre-clinical research for IBD therapeutics that more robustly target the pro-inflammatory GALT. One such area is the use of biomaterials to deliver more targeted therapeutics to the GALT to ameliorate immunological pathogenesis directly.^12^

**Figure 2.**
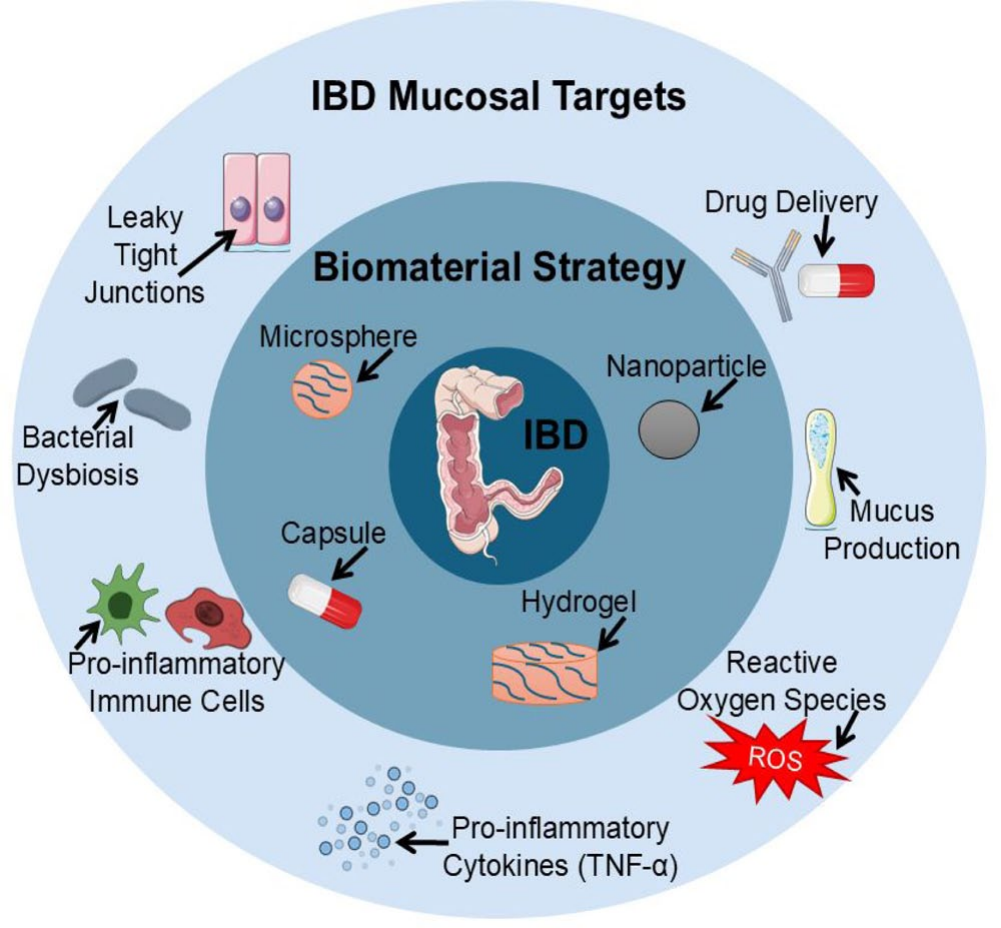
Schematic of mucosal targets specific to an inflamed intestine for a biomaterial therapeutic strategy. Source: Graphics adapted from Servier Medical Art (https://smart.servier.com), licensed under CC BY 4.0 (https://creativecommons.org/licenses/by/4.0/) and OpenScienceArt.

Biomaterials are not a new concept, with first-generation materials being widely used from ~1950s due to being considered bioinert and biocompatible. Since then, biomaterials have progressed to fourth-generation iterations, as engineered smart materials that demonstrate instructive properties with the surrounding biological environment.^13^ Oftentimes, smart biomaterial design stems from bioinspiration, but there is now a movement towards biocooperation, in which the material and biology work together to create novel structures with a wider range of properties.^14^ Biomaterial delivery systems can be as nanoparticles, microspheres or hydrogels derived from natural or synthetic polymers to target the inflamed mucosa (Figure 2). The creation of smart biomaterials manifests the ability to directly address the inflamed GALT in IBD by remodelling using immunomodulatory properties to selectively heal the wounding of the gut directly.^12^

This review outlines advances in the biomaterials field aimed at establishing immunomodulatory materials as a therapeutic alternative to complement existing drug-based approaches for IBD. It examines the various classes of biomaterials currently under investigation, as well as the key translational challenges that must be addressed to enable progression to human trials and the development of biomaterials as a novel therapeutic class for IBD.

## Immunomodulatory biomaterials for IBD

### Immune modulation with naturally derived drug delivery biomaterials

There are numerous naturally occurring polymers that form hydrogels, but the modification and combination of natural polymers with other materials or chemicals is advantageous for fine-tuning biomaterial properties. For instance, one study demonstrated that modifying chitosan with carboxymethyl functional groups, in combination with alginate, enabled the formation of microspheres that facilitate targeted delivery of infliximab to the colon in trinitrobenzene sulfonic acid (TNBS)-induced colitis murine models. Conventional administration of infliximab is associated with the development of anti-tumour necrosis factor-alpha (TNF-α) antibodies, which can lead to a loss of therapeutic response and non-specific immunosuppression following intravenous delivery.^15^ The microspheres promoted the restoration of cellular tight junction proteins, which are essential for maintaining epithelial barrier integrity, and exhibited preferential accumulation at inflamed colonic sites. They also attenuated components of the nuclear factor-κB (NF-κB) signalling cascade and reduced the expression of associated pro-inflammatory cytokines, including TNF-α, CCL2 and IL-1β, at the mRNA level.

The use of natural biomaterials as IBD drug carriers is also demonstrated by a study that created a synthetic mucus from purified porcine mucus combined with anti-TNF-α as an injectable new mucus layer.^16^ This study found that the sequestering of a usual IBD medication in a naturally derived mucus resulted in better retention compared to biologic in solution when intra-rectally administered to TNBS-induced colitis mice. Analysis of anti-inflammatory effects within the mouse model was not tested, but in the presence of lipopolysaccharide-activated monocytic cell lines, the modified mucus decreased in situ TNF-α. The advantage of natural biomaterials in this case is the ability to deliver and retain biologics in the intestines and minimise off-target effects to maximise drug payload at the site of inflammation. This is contrary to the usual intravenous infusion route of biologics, which limits access to the lamina propria and is associated with systemic effects. By demonstrating improved mucosal targeting with already approved IBD therapeutics, biomaterials highlight a delivery method to access the full potential of biologics without the known side effects and off-target effects of systemic administration.

Similar drug delivery repurposing is demonstrated by pectin, another naturally occurring biomaterial. Usually, pectin is highly soluble in the gastrointestinal tract (GIT), but cross-linking pectin with calcium ions forms calcium pectinate (CaP), which shows promise for colonic delivery. CaP nanoparticles were coated with the commercially available synthetic polymer Eudragit S100 to maximise pH-dependent release and were loaded with the immunosuppressant drug azathioprine to test release at a range of pH.^17^ The beads were found to have a maximal release of azathioprine at pH 6.8 by placing coated beads in acidic baths and measuring the release with a UV spectrophotometer. Although demonstrating specific release at colonic pH, the beads were not subjected to cellular or animal testing to determine release in the context of a living environment. Furthermore, the pH of the colon differs from that of an inflamed one, with evidence that active UC reduces luminal pH.^18^ Therefore, this highlights the importance of more thorough testing of specific drug release in the context of colitis to ensure biomaterial translation to the clinic. Another example of natural polymers for IBD biomaterial therapeutics is cellulose due to its known biocompatibility. Ahmad et al.^19^ created an aminocellulose-conjugated polycaprolactone containing Budesonide and coated it onto gelatine-based nanoparticles. These particles acted as vessels to deliver Budesonide directly to an inflamed colon and avoid drug degradation due to pH changes and first-pass metabolism, as well as to minimise side-effects. The particles significantly decreased colonic mast cell infiltration in dextran sodium sulphate (DSS)-induced colitis in mice. There were no observable toxicities or accumulation in off-target organs like the liver, lungs and kidney, suggesting that the nanoparticle optimised drug targeting to the inflamed colon. The results also demonstrated a reduction in TNF-α and IL1-β secretion and downstream inflammatory mediators of inducible nitric oxide synthase and Cyclooxygenase-2 compared to the administration of Budesonide alone. These studies demonstrate the use of natural biomaterials for IBD as drug carriers due to inherent biocompatibility and properties to overcome drug metabolism barriers to maximise the delivery of therapeutic doses.^19^

The use of naturally derived epitopes to enhance nanoparticle efficacy has also been demonstrated by Fan et al.^20^ In this study, the authors synthesised orally poly(ethylene glycol) (PEG) nanoparticles functionalised with butyrate epitopes to enable targeted release of magnolol, a bioactive compound derived from *Magnolia* used in traditional Chinese medicine to modulate the NF-κB pathway. NF-κB is a well-established transcription factor implicated in IBD pathogenesis, exhibiting dysregulated and constitutive activation due to genetic and inflammatory cues.^20^ By optimising butyrate composition, the authors identified formulations that promoted localised magnolol release at inflamed sites, resulting in reduced expression of pro-inflammatory cytokines TNF-α and IL-6, as well as enhanced expression and promoted IEC repair in vitro.^21^ This confers a dual therapeutic function through both drug delivery and intrinsic bioactivity of butyrate. Despite these promising anti-inflammatory effects, the rationale for selecting magnolol over clinically approved IBD therapeutics, such as biologics and small molecules, remains insufficiently justified. Nevertheless, the application of naturally derived polymers with tailored chemical modifications highlights strategies to improve drug delivery to inflamed intestinal tissue by enhancing site-specific bioavailability. These approaches can be further refined by engineering polymers responsive to intestinal cues, including pH and metabolite gradients such as butyrate. Ultimately, the development of biomaterials designed to conjugate or release approved IBD therapeutics may facilitate clinical translation, positioning these systems as adjunctive delivery platforms rather than entirely novel drug classes.

Another strategy to create therapeutic biomaterials is leveraging the inflammatory conditions specific to IBD. One such characteristic is the production of reactive oxygen species (ROS) in the inflamed intestines. ROS are upregulated in IBD due to cellular oxidative stress and changes in the intestinal environment, which further exacerbate inflammation through pro-inflammatory mediator release [IL-1β, IL-6, TNF-α, Interferon gamma (IFN-γ)] and macrophage activation. This is another facet of IBD that adds to the vicious cycle of intestinal inflammation.^22^ In a recent study, natural and synthetic polymers were combined as one modality. The study created a non-invasive pill for IBD diagnostics, due to around 50% of patients not returning stool samples for faecal calprotectin testing and therefore missing out on crucial diagnosis. The authors create an oral pill that releases a blue dye when in contact with ROS, synonymous with gut inflammation, to stain faeces blue.^23^ A 00-sized capsule device was loaded with a brilliant blue dye and was enclosed by a strip of naturally derived dextran polymer. Modified dextran (functionalised with phenylboronic ester groups) lost all integrity within 24 h in the presence of 100 mM hydrogen peroxide (upregulated ROS found in IBD patients). When used to coat the pill, faeces were dyed blue for 78% of DSS-induced colitis rats for 48 h. When the pill was given to the rats sequentially three times, the correct diagnosis increased specificity to 100%. While this work highlights a delivery system that harnesses the inflammatory changes in IBD to allow specific passage through the GIT, it does not demonstrate any changes in the immune environment, including any anti-inflammatory effect via ROS.

Another study instead used thiol-modified hyaluronic acid hydrogels as a biomaterial delivery system to protect probiotics from the harsh GIT.^24^ The study found that areas of inflamed colon with excessive ROS, due to colitis, resulted in hydrogel cleavage and specific release of the probiotics. Not only did this induce anti-inflammatory effects like increased secretion of IL-10 from DSS-induced colitis mice, but the hydrogel also acted as a scavenger for surplus ROS to return to the same levels as the vehicle controls. Another study has demonstrated the use of hydrogels to reduce the production of ROS that are implicated in the progression of IBD.^25^ This was achieved by genetically engineering the probiotic bacteria *Escherichia coli* Nissle 1917 to overexpress the enzymes catalase and superoxide dismutase that reduce inflammation by metabolising ROS. The engineered bacteria were then coated in a mix of the naturally occurring polymers alginate and chitosan, by electrostatic self-assembly, to minimise the breakdown of the probiotic upon oral gavage to DSS-induced colitis mice. The engineered probiotic resulted in significant amelioration of symptoms associated with murine colitis by recovering intestinal barrier integrity and restoring commensal bacteria diversity. Utilising the accumulation of ROS in IBD has also been exhibited as a trigger to initiate gelation of a hyaluronic acid–derived synthetic mucus for intestinal healing.^26^ Mucus is the GIT’s natural hydrogel secreted by goblet cells to protect the epithelium from commensal bacteria. It adheres to the intestinal epithelium surface and maintains structural integrity. Hyaluronic acid was modified with thiol groups to endow the hydrogel with a ROS-responsive nature. 60% thiol substitution in the hyaluronic hydrogel resulted in *E. coli* invasion through the hydrogel. There were no cellular inhibitory effects when the hydrogel was cultured with Caco-2 (IECs) and RAW 264.7 (mouse macrophages) cell lines. When *E. coli* was introduced to the RAW 264.7 culture system, there was an increase in ROS that was then suppressed by the presence of the synthetic hydrogel mucus to the same levels as the phosphate-buffered saline control groups. The biomaterial was demonstrated to form a stable hydrogel in ROS-rich intestines, likely due to the interaction of thiol groups in the gel-forming disulphide bonds with cysteine residues of the native mucus. When administered to colitis-induced mice, the 60% substituted hydrogel diminished mouse weight loss and histological intestinal tissue injury compared to the lower percentage thiol-modified hydrogels and saline vehicle mice. Furthermore, the synthetic mucus downregulated the recruitment of M1 macrophages and associated inflammatory cytokines whilst promoting CD206-expressing macrophages and associated anti-inflammatory cytokines to the mouse colon. The authors created a chronic colitis mouse model with intermittent administration of DSS over 50 days. When treated with the 60% thiol-modified hydrogel, the mice had the lowest IBD-related symptoms compared to lower percentage thiol-modified hydrogels and saline controls due to the re-establishment of microbiota gut diversity.

A similar mucus-like hydrogel has also been demonstrated by a study that created hyaluronic acid that was again modified by thiol groups to form a hydrogel in a murine colitis model via thrombin-mediated fibrinogen activation.^27^ The hydrogel was directly administered to the colon by rectal enema and created a hydrogel plug at sites of inflammation. The acidic UC environment of the intestine resulted in the gradual degradation of the artificial mucus plug to release silver ions, which have antibacterial properties and eliminate harmful bacteria to restore commensal homeostasis. The fibrin network left after degradation also served as an anti-inflammatory unit that repolarised M1 macrophages to M2 via toll-like receptor 4 and suppression of NF-κB signalling. This demonstrates that the creation of a natural hydrogel combines specificity towards ROS for selective delivery of therapeutics in IBD animal models, but also harnesses the intestine’s natural architecture, like the epithelial mucus layer. This facilitates close interactions between the biomaterial and inflammatory environment to elicit anti-inflammatory effects as a more selective therapy for IBD.

One approach through which natural hydrogels can exploit the intestine’s intrinsic composition is with decellularised matrices. In one such study, a hydrogel derived from porcine intestine was generated via decellularisation, producing a matrix enriched with native biomolecules such as collagen, proteoglycans, and growth factors.^28^ The collagen component facilitated hydrogel formation while also serving as a delivery vehicle for mesenchymal stem cells (MSCs) in DSS-induced colitis murine models. This approach resulted in reduced expression of pro-inflammatory markers and improved epithelial barrier integrity. MSC-based therapies have advanced to clinical trials largely due to their immunomodulatory properties, particularly through the secretion of trophic factors that promote regulatory Treg expansion and support mucosal repair. However, systemically administered MSCs frequently exhibit off-target accumulation in organs such as the spleen and liver, thereby limiting their therapeutic efficacy at sites of intestinal inflammation. The use of a naturally derived hydrogel matrix, therefore, represents a promising strategy to enhance therapeutic localisation and maximise immunomodulatory effects within inflamed tissue. Advancing these combinatorial approaches towards clinical translation could improve the targeting and overall efficacy of MSC-based therapies, further strengthening their potential as a treatment for IBD.

There is a clear movement towards natural polymers as targeted drug delivery systems specific to the IBD environment to maximise immune modulation in the form of nanoparticles and hydrogels. The advantage of naturally derived biomaterials is the abundance in nature, which would facilitate long-term accessibility of raw materials for drug production. Natural biomaterials usually have known biocompatibility and biodegradability due to already existing awareness and knowledge of the materials. Future research using naturally derived biomaterials should incorporate chemical modification testing to understand the added benefits of altered biomaterials, as well as long-term testing periods with *in vivo* and *in vitro* models to discount biomaterials that would not be compatible with living systems. To add to this, using more advanced models that better bridge the gap between pre-clinical and clinical testing, whether *in vitro, in vivo or ex vivo*, would be the next step in biomaterial research for IBD. This would build further in-depth evidence of biomaterial properties and effects in a more human-like context to aid the selection of safe candidates for clinical trials, as an area missing from current research (Tables 1–3).

**Table 1.** Summary of naturally derived biomaterials and experimental outcomes.

Biomaterial class	Biomaterial properties	Immunomodulatory IBD target	Model type	Immunomodulatory effect	
Microspheres	Chitosan with carboxymethyl modifications with alginate	Infliximab loaded for targeted anti-TNF-α	Colitis mice	↑ Cellular tight junctions	[15]
				↓ NF-κB	
Nanoparticles	Calcium pectinate-pectin crosslinked with calcium ions and coated with Euragit	Azathioprine release from nanoparticles	None	n/a	[17]
Hydrogel	Methylated cellulose	Delivery of drugs like Budesonide and glycyrrhizic acid	Colitis mice	Glycyrrhizic acid delivery	[29]
				↓ Mucosal inflammation	
Nanoparticles	Aminocellulose-conjugated polycaprolactone	Budesonide delivery, contained within a nanoparticle	Cell lines	↓ Mast cell infiltration	[19]
				↓ ROS	
				↓ TNF-α	
Nanoparticles	Butyrate-rich polymer	Delivery of natural anti-inflammatory magnolol to colonic tissue	Colitis mice	↓ TNF-α	[21]
				↓ IL-6	
				↓ ROS	
			Cell lines	↑ Intestinal epithelium healing	
Polymer-coated capsule	Modified dextran polymer, sensitive to ROS	Release of blue dye in the inflamed intestines to indicate colitis	Colitis rats	ROS detection	[23]
Engineered probiotic coated with a polymer	Alginate and chitosan polymer coating to minimise bacterial degradation	Engineered bacteria designed to metabolise ROS	Colitis mice	↑ Intestinal epithelium healing	[25]
				↑ Intestinal bacteria diversity	
Hydrogel	Thiol-modified hyaluronic acid hydrogel	ROS triggered gelation-sensitive hydrogel to embed with native mucus to restore the mucosal barrier	Colitis mice	↓ ROS	[26]
			Cell lines	↓ M1 macrophages	
				↑ M2 macrophages	
				↑ Intestinal bacteria diversity	
Hydrogel	Thiol-modified hyaluronic acid hydrogel sequestered with silver ions and activated via thrombin-mediated fibrinogen gelation	Creation of a hydrogel plug in the intestines that degraded in an acidic environment to release antibacterial silver ions and repolarise macrophages via the remaining fibrin network	Colitis mice	↑ Intestinal bacteria diversity	[27]
				↓ M1 macrophages	
				↑ M2 macrophages	
				↓ NF-κB	

IBD, inflammatory bowel disease; NF-κB, nuclear factor-κB; ROS, reactive oxygen species; TNF-α, tumour necrosis factor-alpha.

**Table 2.** Summary of synthetically derived biomaterials and experimental outcomes.

Biomaterial class	Biomaterial properties	Immunomodulatory IBD target	Model type	Immunomodulatory effect	
Nanoparticles	Poly(lactic-co-glycolic) acid + poly(ethylene glycol) loaded with *Ramulus mori* polysaccharide.	Inflammation + mucus-penetrating.	Colitis mice	↑ IL-10 + SCFAs	[30, 31]
			Cell lines	↓ IL-6, IFN-γ + TNF-α	
Nanoparticles	Chitosan modified Poly(lactic-co-glycolic) acid loaded with *Phellinus igniarius* polysaccharide.	M1 macrophages + mucus-penetrating.	Colitis mice	↑ SCFAs	[32]
				↑ Intestinal bacteria diversity	
			Cell lines	↓ M1 macrophages	
Nanoparticles	Eudragit modified poly(lactic-co-glycolic) acid loaded with garcinol	Inflammation	Colitis mice	↓ NF-kB, TNF-α + IL-8	[33]
			Cell lines		
Microspheres	Collagen modified poly(lactic-co-glycolic) acid with exterior mesenchymal stem cells.	Paracrine activity between stem cells and pro-inflammatory IBD cells.	Colitis mice	↓ IFN-γ + T cells.	[34]

IBD, inflammatory bowel disease; NF-κB, nuclear factor-κB; ROS, reactive oxygen species; SCFAs, Short-chain fatty acids; TNF-α, tumour necrosis factor-alpha.

**Table 3.** Summary and comparisons of properties of conventional and supramolecular polymers.

Property	Conventional polymers	Supramolecular self-assembling polymers
Bonding	Covalent	Non-covalent
Curing	Chemical, heat, radiation	pH, hydrophobic collapse, metal ion coordination, steric repulsion
Source	Natural + synthetic	Natural + synthetic (often inspired or assembled with biomolecules)
Biocompatibility	Requires consideration during synthesis + curing	Requires consideration in terms of structural reversibility and potential degradation release
Example biomaterial classes	Hydrogels, Nanoparticles	Micelles, nanoparticles, hydrogels
Examples	Alginate, gelatine, PLGA, PEG	Peptide amphiphiles, DNA

PEG, poly(ethylene glycol); PLGA, poly(lactic-co-glycolic) acid.

### Immune modulation with synthetic drug delivery biomaterials

Like biomaterials derived from naturally occurring polymers, there is also research into immunomodulatory therapeutics developed from synthetic biomaterials to engineer IBD drug carriers. Synthetic biomaterials offer more effective immune modulation due to the easier tuneability of polymers to withstand delivery in the harsh GIT environment. The most notable synthetic polymer is poly(lactic-co-glycolic) acid (PLGA) due to being highly biocompatible and biodegradable into the physiological substrates lactic and glycolic acid.^30^ In a recent study, *Ramulus mori* polysaccharide was loaded into PLGA nanoparticles to have various localised effects on DSS-induced colitis mice.^31^ The mice had significantly less secretion of IL-6 and IFN-γ and increased IL-10 and short-chain fatty acid production when treated with the PLGA-polysaccharide system. This work supports PLGA as an effective drug carrier but also as an adjuvant for downregulating pro-inflammatory phenotypes when used in combination with other therapeutics. Similarly, PLGA nanoparticles conjugated to either 2 or 5 kDa PEG chain and were tested as delivery systems for encapsulated anti-TNF-α antibodies in both an in vitro and in vivo setting.^32^ The study proposes that the use of PLGA is advantageous for reasons previously discussed and that PEGylated nanoparticle surfaces have been previously shown to accumulate at inflamed sites and be mucus-penetrating. The particles were found to be biocompatible and to be taken up by cell lines, and, when loaded with the antibody, showed a concentration-dependent decrease in secreted TNF-α. When the nanoparticles were administered to DSS-induced colitis mice, only the 2 kDa PEGylated surface had significant anti-inflammatory effects in terms of reduction in tissue-associated myeloperoxidase activity and TNF-α. The authors suggest that due to the longer PEG chain having a higher hydrophilicity, the nanoparticle is degraded more quickly and the anti-TNF-α antibody is released earlier to have a significant anti-inflammatory effect. The use of PLGA as a drug carrier is evident, as it is an adaptable and biocompatible polymer.

Like natural polymers, combining synthetic delivery systems with other biomaterials enhances their properties. Like the *Ramulus mori* polysaccharide PLGA nanoparticles, *Phellinus igniarius* polysaccharide was loaded into PLGA nanoparticles that had been modified with chitosan, due to its mucoadhesive properties, to enhance intestinal absorption.^33^ The administration of the nanomedicine to DSS-induced colitic mice resulted in reduced M1 macrophage polarisation, as the prominent cell type promoting inflammation in IBD, and increased short-chain fatty acids to re-establish gut microbiota. Another example is the coating of PLGA nanoparticles with Eudragit, which greatly increased the pH responsiveness with specific release of Garcinol (anti-inflammatory molecule derived from fruit) at pH 7.4 to suppress NF-kB, TNF-α and IL-8 in both and in vitro and in vivo settings.^34^ A more sophisticated method to functionalise particle delivery systems is demonstrated by the modification of PLGA particles with an exterior coating of extracellular matrix proteins like collagen. More specifically, this methodology was demonstrated by the attachment of MSCs to the exterior of microspheres via a collagen coating, with the collagen attached to the PLGA sphere via conjugated dopamine. As stated previously, exogenous mesenchymal stem cell therapy shows promise against inflammatory conditions like IBD due to paracrine interactions between implanted stem cells and host cells, but often lacks efficacy due to poor survival of the cells after transplantation.^35^ The addition of collagen-binding sites to the microspheres demonstrated enhanced cellular viability during intraperitoneal administration to DSS-induced murine colitis. When the mesenchymal stem cell microspheres were delivered to colitic mice, the enhanced cellular viability allowed for amelioration of symptoms from reduced IFN-γ secretion to inhibited CD4+ and CD8+ T cells infiltrating into the colon.^36^ Similarly, microspheres formed of PLGA and chitosan were functionalised with E- and N-cadherin motifs to stem cell attachment, like the previously described dopamine-conjugated collagen.^37^ As well as controlled release of IL-1β to prime the cells for activation. As before, the functionalisation of microspheres created a biomaterial able to secrete anti-inflammatory mediators (like TGF-β and IL-10) from the attached MSCs in the same pre-clinical models used before. Although an exciting concept, more work is needed to determine the exact mechanism of action of MSCs delivered by intraperitoneal injection at the site of inflammation. Colitis alleviation may be due to secretory factors from the stem cells or contact-dependent interaction of inflammatory host cells and implanted stem cells, as well as the long-term effects of mesenchymal stem cell injections. Understanding this therapeutic mechanism would facilitate movement towards clinical trials for biomaterial-based stem cells as an IBD treatment.

Polymer-based biomaterials can act as carriers of immunomodulatory therapeutics or have direct anti-inflammatory effects. However, traditional synthetic polymer biomaterials are formed by covalent interactions that result in a fixed structure and therefore functionality due to the structure being predetermined by monomer unit bonding.^38^ Oftentimes, covalent polymers rely on curing methods involving ultraviolet light or chemical crosslinkers to cement the polymeric structure. This limits biocompatibility due to curing occurring before applying to a biological system or interfering with the activity of therapeutics carried by the polymer.^39^ Therefore, the design principle of ‘bottom-up’ fabrication to form complex polymer-like structures through non-covalent self-assembly bolsters more opportunities for biomaterial design.^40^ This methodology uses molecular building blocks to assemble into precise supramolecular three-dimensional structures by drawing upon the principles of biological self-assembly.^41^ Unlike traditional polymers, supramolecular self-assembly occurs via non-covalent interactions and results in structures that showcase higher complexity in terms of molecular design, presentation and specificity due to the biomaterial not being reliant on fixed covalent chemical bonds.^38,42^ This enables the supramolecular polymer to be dynamic and to undergo rapid structural changes in response to the biological milieu.^40^ The advantages of supramolecular polymers for therapeutic delivery are well defined. With a major therapeutic benefit being reduced biological rejection associated with the foreign body response, which is often prescribed to unfavourable polymer surface chemistry that initiates a pro-inflammatory reaction against the material.^43^

**Figure 3.**
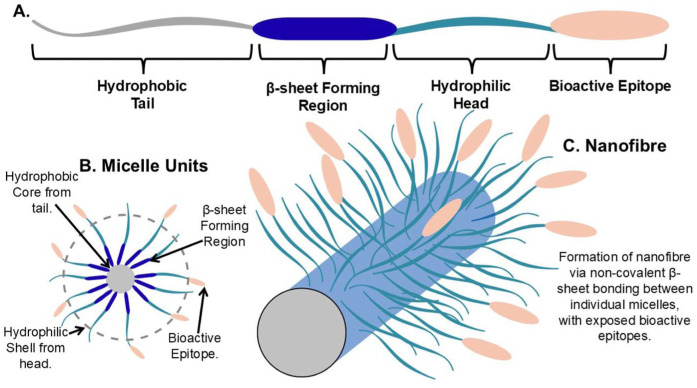
Schematic of peptide amphiphile structural units and assembly. (a) Each peptide amphiphile unit has distinctive regions to facilitate the creation of (b) micelle units and (c) nanofibrous hydrogels in aqueous conditions and display bioactive epitopes to the external environment. Graphics made in canva.com.

**Figure 4.**
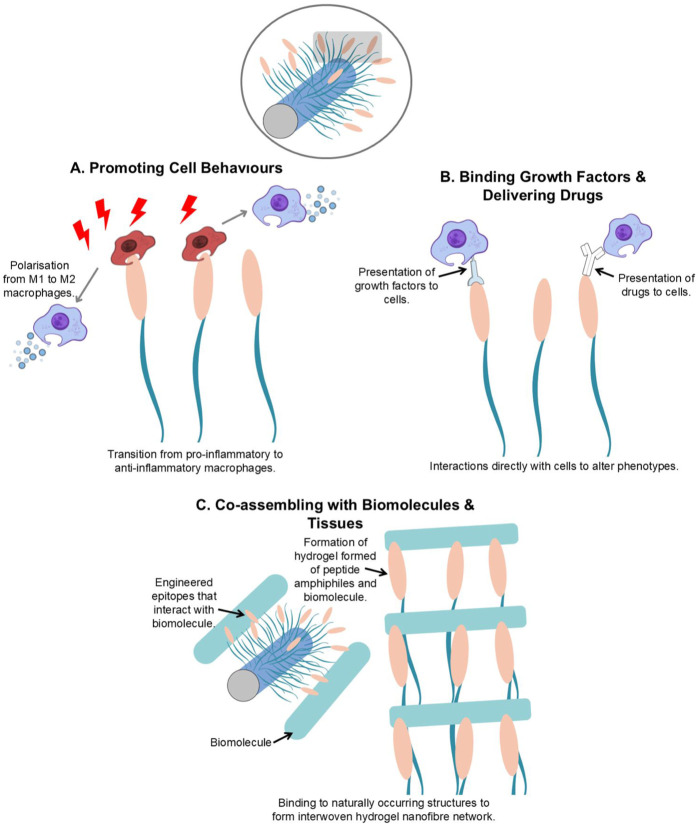
Peptide amphiphiles demonstrate tuneability and bioinstructive properties due to the ability to engineer the bioactive epitopes that are exposed to the external environment. This allows the epitope to directly interact with its biological surroundings to have an immune-modulatory effect. This may be achieved by repolarisation or cells (a) or influencing cell behaviour through presentation of phenotype altering drugs or growth factors (b), or by creating new biomaterials via interactions with surrounding biomolecules (c). Source: Graphics made in canva.com.

Peptide amphiphiles (PAs) are an example of a supramolecular biomaterial. PAs demonstrate amphiphilic properties to enable polymer self-assembly into highly tuneable and biologically compatible hydrogels, as determined in the seminal paper of Samuel Stupp.^44^ Supramolecular assembly relies upon weak non-covalent interactions between monomers to form structural stability as a whole unit.^40^ A PA monomer structure has three distinct regions: a hydrophobic alkyl tail of at least 10 carbons; β-sheet forming peptide region and a charged peptide region designed to act as the polar head of the molecule created by solid phase peptide synthesis^44,45^ (Figure 3). In an aqueous environment, PA self-assembly is driven by hydrophobic interactions between hydrocarbon tails along with hydrogen bonding facilitating the creation of β-sheets to form well-defined nanofibres (Figure 3). PAs can also include bioactive epitopes^46^ connected to the polar head and displayed on the surface of the nanofibres to interact with the environment (Figure 4), for example, promoting cell behaviours^47^ (Figure 4), binding growth factors,^44^ delivering drugs^39^ (Figure 4) or tuning their mechanical properties.^48^ Furthermore, multiple PAs can be assembled to generate nanofibres displaying multiple signals.^49^ In addition, PAs can be designed to co-assemble with extracellular matrix components such as proteins^50–52^ or polysaccharides,^53,54^ opening the opportunity to assemble and integrate with tissues or biological fluids in situ (known as biocooperation^55,56^; Figure 4).

The therapeutic potential of PAs is due to biocompatible, biocooperation and tuneable and pro-healing properties.^56–58^ In a recent study, Bury et al. demonstrated novel anti-inflammatory PAs (Tx-PA), that reduced the number of pro-inflammatory cells associated with IBD and related cytokines in the CD-like ileitis mouse model known as SAMP1/YitFcJ.^59^ The Tx-PAs display a small peptide derived from uteroglobin protein, which modulates inflammation produced by leukocytes that extravasate to intestinal tissue by regulating the expression of adhesion molecules like L-selectin found on the cells. After structural confirmations of PA self-assembly, the researchers injected Tx-PA, saline or scrambled PA sequence (vehicle) directly into the inflamed areas of the intestines of SAMP1/YitFcs mice. The intestines were placed back into the abdomen, and the cavity was sutured. Treated ileum lesions were collected 15 weeks after the operation to assess inflammatory cellular and cytokine markers using immunohistochemistry. Data were produced by immunofluorescent quantification of stained samples and showed that Tx-PA reduced CD68+, CD4+, CD11c, mast cell tryptase, TNF-α, IFN-γ, IL-13, CD86+ macrophages and increased IL-10 and CD206+ macrophages in the Tx-PA-treated mice. This paper assesses anti-inflammatory PA in the context of ileitis and boasts a true reversal in the inflammatory phenotype of Crohn’s-like ileitis. Despite the major surgery required to deliver these PAs, the direct administration to inflamed areas reduced the likelihood of the Tx-PA being absorbed in the stomach before entering the intestinal tract and to minimise systemic effects of administration against TNF-α and IFN-γ, unlike current biologic options for IBD.

As previously mentioned for naturally derived biomaterials, the GIT’s natural polymer hydrogel, mucus, can be used as a specific IBD architecture for biomaterial incorporation via non-covalent interactions and covalent cross-linking. These principles were leveraged to create an ‘ultrastable mucus-inspired hydrogel’ (UMIH), tested in the context of oesophageal mucosal healing.^60^ The UMIH addresses conventional hydrogel limitation of stability under acidic conditions. The UMIH was developed using high-isoelectric point proteins ELR-IK24 that are abundant in basic amino acids. When in a low pH environment, the basic amino acids become protonated to increase electrostatic interactions with tannic acid that enhances wet adhesion via hydrogen bonding and hydrophobic interactions to facilitate hydrogel structural integrity, along with the crosslinked hexamethylene diisocyanate (HDI) that prevents acid-induced degradation for overall stability in acidic environments. The structural characteristics of UMIH were shown to be a dense, nanofibrous hydrogel structure with significantly higher storage modulus readouts compared to controls. The UMIH is shown to be a shear-thinning material, essential for injectability and structural recovery when delivered in vivo. Furthermore, using acidic conditions (pH 2.0), the UMIH was measured to have a strength of 64.7 kPa under shear adhesion testing and maintained a stable mass over 7 days in acidic solution. UMIH has been shown to be non-toxic and enhance cellular proliferation when applied to oesophageal epithelial and macrophage cell lines in vitro. Macrophages demonstrated a shift towards M2 phenotype, shown by messenger RNA levels for CD86 and CD206. When implanted in porcine oesophageal tissue, UMIH demonstrated reduced leukocyte recruitment and an increase in angiogenic markers from immunohistochemical tissue staining. Lastly, the UMIH was established as being disruptive to bacterial pathogen membranes when imaged by scanning electron microscopy with *E. coli* and *Staphylococcus aureus*. The creation of this UMIH leverages the natural properties of in situ mucus and demonstrates antimicrobial, anti-inflammatory and pro-healing properties of the hydrogel in cellular and animal models. More rigorous biocompatibility testing is essential for progression past pre-clinical testing, but combining natural mucosal hydrogel properties with chemical adjustments evidences the advantages of hydrogels as materials that can easily mirror biological properties to be directly integrated into the intestinal system to elicit immunomodulatory effects.

The use of synthetic-based biomaterials poses an advantage over naturally derived ones in that they allow for the ability to fully tune the material properties from scratch. This is even more apparent with supramolecular self-assembling polymers compared to conventional ones, due to the ability to biocooperate and mimic natural structures of the intestinal mucosa. But, like, naturally derived polymers, the integration with already approved polymers like Eudragit enhances the delivery and druggability of immunomodulation to the intestines.

## Conclusion

A key limitation of current IBD therapies is their reliance on broad immunosuppression to achieve clinical remission, rather than addressing the underlying causes to enable durable disease resolution. This review highlights the therapeutic potential of biomaterials, particularly their tuneability and capacity for targeted delivery to inflamed GALT, thereby directly addressing the central driver of IBD immunological dysregulation. A notable feature of emerging biomaterial strategies is the design of systems that respond to specific inflammatory cues, enabling stimulus-triggered therapeutic action and minimising systemic and off-target effects commonly associated with conventional treatments. A major distinction between synthetic and natural biomaterials lies in the ability to engineer synthetic polymers de novo with tailored physicochemical properties for specific functions within the GIT. Although natural polymers are abundant, renewable, and inherently biocompatible, synthetic supramolecular polymers facilitate in situ material formation within the body through biocooperation, enabling dynamic interactions with biological systems. This property offers a distinct advantage, allowing biomaterials to integrate with physiological structures and potentially form functional constructs that modulate immune responses directly at sites of inflammation. While further investigation is required to characterise long-term safety and stability, the incorporation of biomaterials that interface with naturally renewing components, such as mucus, may provide predictable degradation pathways. Looking forward, biomaterial research in IBD should continue to leverage intestinal physiology and inflammatory cues to guide the design of targeted delivery systems. Furthermore, positioning biomaterials as adjuncts to existing therapeutics – rather than standalone drug classes – may accelerate clinical translation by aligning material innovation with established treatment paradigms.

Amongst current translational gaps between immunomodulatory biomaterial research to the clinic are the platforms used for therapeutic testing to progress research from the bench to the bedside. Common platforms discussed in this review rely on DSS-induced colitis animals as well as cell lines. IBD is an extremely complex disease with multifactorial aetiology and symptom presentation. These factors are yet to be fully recapitulated in therapeutic models, but creating a biomaterial that directly addresses the complex intestinal immune system is integral to move away from conventional biologics and corticosteroid intervention. This translational gap becomes more apparent with the UK government’s roadmap to phase out animal testing with scientific investment for alternative models. Even though DSS-induced mice do not properly recapitulate IBD in humans, it does allow examination of off-target accumulation of drugs from the intestines compared to cellular models. To add to this, due to the chronic nature of IBD and periods of remission and flare-up, the effects of immunomodulatory biomaterials require testing in models of heightened and reduced inflammation to create drug options for the pathophysiological changes of the disease. Future biomaterial research requires platforms in complex cellular models to meet the demands of rigorous pre-clinical testing and complies with UK legislation. Advancing the platforms used to test immunomodulatory biomaterials at the pre-clinical stage would create a broader body of evidence to select biomaterial candidates for clinical trials and to understand therapeutic benefits in patients.

Immunomodulatory biomaterials, whether natural or synthetic, have been demonstrated as beneficial in various publications with success in overturning inflammatory markers of IBD in pre-clinical models. Interdisciplinary collaborations are essential to delineate the current limitations of IBD-specific biomaterials to emerge as a realistic therapy. The real advantage of biomaterials is the ability to engineer properties for targeted IBD treatment. This has taken the form of adjuvants to repurpose current therapeutics or respond and integrate with physiological features of the GIT in situ for targeted mucosal delivery.

## Methods

A literature search was conducted using PubMed, Google Scholar and Web of Science databases. The primary aim was to identify pre-clinical, laboratory studies that investigated biomaterials in the presence of IBD models.

The screening process started with a search for ‘Immunomodulatory Biomaterials IBD’.

Papers were extracted and compared to assess themes from the literature. Given the broad scope and diverse data in this review, a narrative synthesis was conducted to identify, summarise, and explore research areas, avoiding duplication. Narrative reviews address multiple questions but can lack clear inclusion criteria and risk bias due to subjectivity in study selection.

## Declarations

**Ethics approval and consent to participate:** Not applicable.

**Consent for publication:** Not applicable.

**Availability of data and materials:** Not applicable.
